# Long-term impact of the expansion of a hospital liaison psychiatry service on patient care and costs following emergency department attendances for self-harm

**DOI:** 10.1192/bjo.2020.18

**Published:** 2020-04-02

**Authors:** Joni Jackson, Manjula D. Nugawela, Frank De Vocht, Paul Moran, William Hollingworth, Duleeka Knipe, Nik Munien, David Gunnell, Maria Theresa Redaniel

**Affiliations:** The National Institute for Health Research Applied Research Collaboration West (NIHR ARC West), University Hospitals Bristol NHS Foundation Trust; and Population Health Sciences, Bristol Medical School, University of Bristol, UK; Bristol Medical School, University of Bristol, UK; Centre for Academic Mental Health, Population Health Sciences, Bristol Medical School, University of Bristol, UK; Population Health Sciences, Bristol Medical School, University of Bristol, UK; University Hospitals Bristol NHS Foundation Trust, UK

**Keywords:** Self-harm, liaison psychiatry service, emergency department

## Abstract

**Background:**

In September 2014, as part of a national initiative to increase access to liaison psychiatry services, the liaison psychiatry services at Bristol Royal Infirmary received new investment of £250 000 per annum, expanding its availability from 40 to 98 h per week. The long-term impact on patient outcomes and costs, of patients presenting to the emergency department with self-harm, is unknown.

**Aims:**

To assess the long-term impact of the investment on patient care outcomes and costs, of patients presenting to the emergency department with self-harm.

**Method:**

Monthly data for all self-harm emergency department attendances between 1 September 2011 and 30 September 2017 was modelled using Bayesian structural time series to estimate expected outcomes in the absence of expanded operating hours (the counterfactual). The difference between the observed and expected trends for each outcome were interpreted as the effects of the investment.

**Results:**

Over the 3 years after service expansion, the mean number of self-harm attendances increased 13%. Median waiting time from arrival to psychosocial assessment was 2 h shorter (18.6% decrease, 95% Bayesian credible interval (BCI) −30.2% to −2.8%), there were 45 more referrals to other agencies (86.1% increase, 95% BCI 60.6% to 110.9%) and a small increase in the number of psychosocial assessments (11.7% increase, 95% BCI −3.4% to 28.5%) per month. Monthly mean net hospital costs were £34 more per episode (5.3% increase, 95% BCI −11.6% to 25.5%).

**Conclusions:**

Despite annual increases in emergency department attendances, investment was associated with reduced waiting times for psychosocial assessment and more referrals to other agencies, with only a small increase in cost per episode.

Self-harm causes a significant international public health burden and is a key risk factor for deaths because of suicide.^1–4^ In England, around 200 000 people present to emergency departments annually because of self-harm and more than 15% present again with repeated self-harm within a year.^5,6^ The direct and indirect costs associated with self-harm are considerable.^7–11^ Providing effective, evidence-based clinical care could reduce repeated self-harm attempts and suicide.^2,12^ UK guidelines recommend that all patients presenting to healthcare because of self-harm should be offered a psychosocial assessment.^13^ The recommended assessment includes an evaluation of the factors leading to self-harm and a full mental health and social needs assessment.^13^ Despite this, the provision of care remains highly variable across UK hospitals.^14–16^

Liaison psychiatry services (LPS) provide care for patients presenting to emergency departments with mental health problems and those who have developed mental health problems while being treated in hospital.^17^ LPS provide psychiatric assessment and treatment to general hospital patients. These include those attending emergency departments as well as in-patients. A recent study of an expanded LPS in a large acute hospital in Birmingham indicated that it led to a reduction in bed use with overall benefit to cost ratio of 4:1.^17^ In September 2014, Bristol Clinical Commissioning Group commissioned University Hospitals Bristol NHS Foundation Trust to expand the operating hours of the LPS at a large teaching hospital (Bristol Royal Infirmary, BRI). The working hours of the LPS service in BRI were extended from Monday to Friday 9.00–17.00 h (40 h per week) to 7 days a week 8.00–22.00 h (98 h per week). In previous work we have demonstrated that the LPS expansion led to several initial improvements in the management and outcomes of patients who have self-harmed.^18^ These included an increase in the proportion of self-harm attendances in the emergency department that received a psychosocial assessment, a reduction in median waiting time for an assessment and a reduction in the proportion of patients self-discharging without a psychosocial assessment.^18^ However, these results were based on a short-term comparison of 3 months before and 3 months after the LPS expansion. Current evidence on the long-term impact of LPS expansion remains limited. This study aims to assess the impact of the £250 000 annual investment to expand the LPS in BRI on the outcomes of self-harm attendances, psychosocial assessments, repeat attendances and treatment costs for emergency department attendances for self-harm, over a 3-year period prior to and after service expansion. The findings will inform commissioners of the potential benefit of investing in LPS.

## Method

We used Bayesian structural time-series methods^19,20^ to evaluate the long-term impact of the expansion of LPS in Bristol, using anonymised data of patients presenting to the BRI emergency department following self-harm between 1 September 2011 and 30 September 2017. The study period was selected to cover a 3-year period before and after the LPS expansion beginning around September 2014.

### Data

Data for all self-harm attendances at the emergency department were obtained from the Bristol Self-Harm Surveillance Register (BSHSR). The BSHSR is a database maintained in the emergency department of BRI that records detailed information on patients presenting to hospital for self-harm since 2010.^21^ Self-harm is defined in the BSHSR as intentional self-injury or self-poisoning irrespective of motivation or degree of suicidal intent. The BSHSR includes clinical and sociodemographic details of all hospital-presentations for self-harm. Data collection is approved by the Central Bristol Research Ethics Committee.

Monthly summary data for each outcome were calculated to investigate the effect of the investment on the total number of episodes admitted to a hospital ward (observation, intensive therapy unit (ITU) or other general ward), the total number of self-harm episodes that received a psychosocial assessment, the total number of episodes self-discharging from the emergency department without a psychosocial assessment, the total number of patients who had a repeat emergency department attendance within 6 months of their index attendance (a patient's first self-harm attendance during the study period with no recorded self-harm attendance within the 6 months prior), the median waiting time (hours) from arriving in the emergency department to receiving a psychosocial assessment, the total number of referrals made to other agencies (including crisis, alcohol, self-harm, social services), and service costs. Psychosocial assessment costs were calculated according to the profession of the assessor, assuming a 90 min assessment using an average salary of a band 7 nurse (liaison nurse) or a weighted (80:20, reflecting the involvement of staff of different grades in assessments) average of junior (foundation year 1 and 2) and senior (registrar and consultant) doctor's salaries, plus overheads, oncosts and indirect time (see supplementary Table 1 available at https://doi.org/10.1192/bjo.2020.18 for cost breakdown).^22^ National Health Service (NHS) reference costs,^23^ stratified by whether the patient was subsequently admitted to hospital (£221.25) or not (£127.56), were used to estimate the costs of emergency department attendance. The average unit cost (£366.56) for a non-elective short-stay admission for ‘observation and counselling’ was used as a proxy for the daily cost of observational unit or other ward care and the average daily cost (£1250.69) of adult medical critical care patients was used to estimate costs for ITU days. Net hospital costs were calculated as the sum of the average psychosocial assessment, emergency department, observation and ITU costs each month.

Monthly averages were also obtained for covariates assumed to be unaffected by the investment, to be used in constructing a control time series predicting what would have happened to the outcomes if the investment had not happened: total number of attendances for self-harm, mean age of patients attending for self-harm and the proportion of patients attending for self-harm that were female.

### Statistical methods

Structural time series models,^19,20,24^ in combination with Bayesian spike and slab regression and Bayesian model averaging, were used to estimate the impact of the expansion of the LPS in Bristol on each outcome. To describe what would have happened had the investment not been provided, a control time series (counterfactual) for each outcome was modelled using the measured pre-investment (1 September 2011 to 31 July 2014) data and the pre- and post-investment time trends of the covariates described above.^20,25,26^ The implementation date of July 2014 was selected in order to avoid incorporating potential anticipatory effects shortly prior to the expansion of the LPS that came into effect in September 2014, such as the recruitment of liaison nurses. The post-investment period was 1 August 2014 to 30 September 2017.

Each outcome was modelled using a first-order autoregressive (AR(1)) process, whereby the value of the outcome at each time point in the series is based on the value of the outcome at the immediately preceding time point taking into account random noise. A seasonal component was included to account for monthly fluctuations. See online supplementary material for model specifications. The impact of the investment was then estimated by comparing the observed time series of the measured outcomes during the post-investment period with the control time series.

The model assumes that any existing relationship between the time series of the covariates and the outcomes remained the same before and after the expansion of the LPS. This was confirmed by a sensitivity analysis (data not shown). Model fit was assessed using Geweke diagnostics, Raftery–Lewis diagnostic tests, mean absolute 1-step prediction errors, Durbin–Watson test, Ljung–Box test and autocorrelation plots.

Bayesian structural time series were constructed using the bsts package in R,^27^ and subsequently used as input for the Causal Impact R package.^28^ Results are presented as monthly averages over 3 years. Relative effects are presented as point estimates of the difference between the average observed and predicted values post-intervention as a percentage of the predicted value post-intervention, with Bayesian 95% credible intervals (BCIs; the interval within which the true value falls with 95% probability, given the model and the data^29^). Posterior predictive *P*-values are calculated and interpreted as the posterior of the mean of classical *P*-values.^30^

## Results

**Fig. 1:**
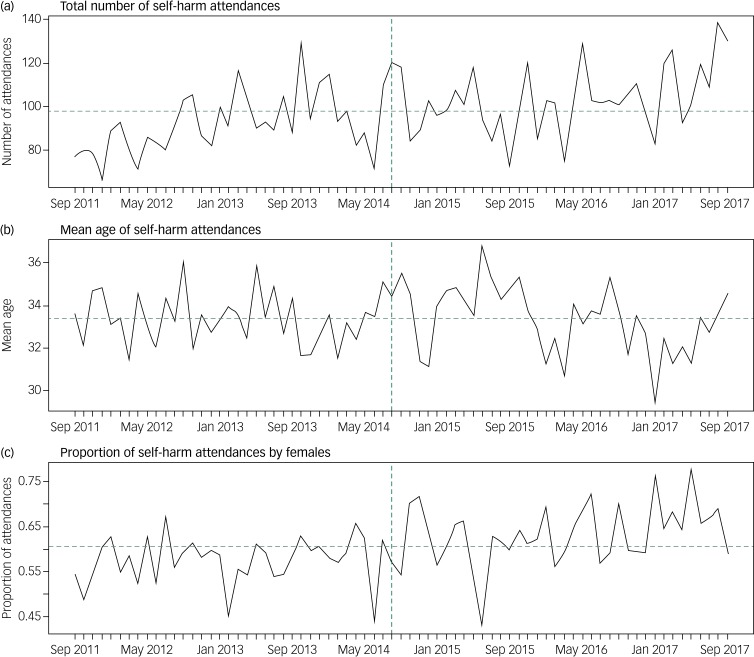
Total number, mean age and proportion of female attendances of self-harm per month between September 2011 and September 2017.

**Fig. 2:**
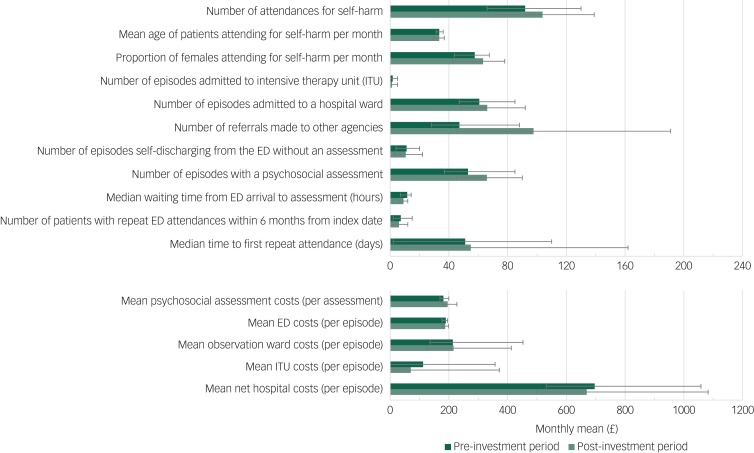
Monthly mean values of covariates and outcomes in the pre-investment period (dark green) and post-investment period (light green).

The temporal trends of the covariates (Fig. 1) indicate an overall increase in the mean number of attendances for self-harm (12 attendances) every month and the proportion of self-harm attendances made by females (5.7%) in the post-investment period compared with the prior period. Mean age of all self-harm attendances in the post-investment period remained the same as that observed in the pre-investment period. The mean and range of each covariate and outcome in the time period before and after the investment are presented in supplementary Table 2 and graphically in Fig. 2. Comparing pre-investment and post-investment means: the mean number of self-harm attendances increased 13%, the median waiting time from arrival in the emergency department to receiving a psychosocial assessment decreased from 11.6 h to 9.0 h, the number of referrals made to other agencies increased from 47.1 to 97.6, the number of episodes that received a psychosocial assessment increased from 53.0 to 65.8 and net hospital costs decreased from £696.30 to £669.30.

Error bars represent the range. ED, emergency department.

**Fig. 3:**
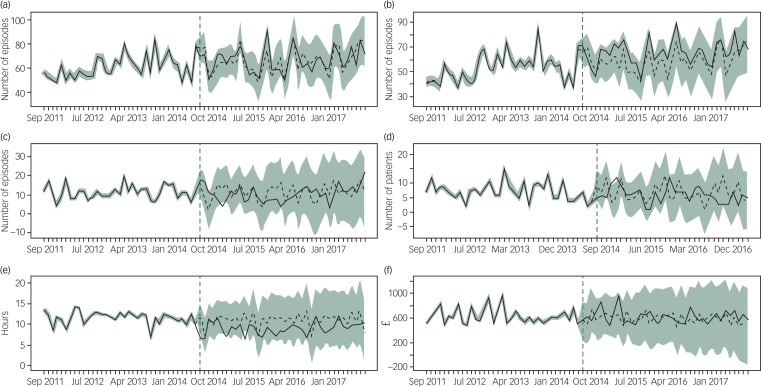
Observed (solid line) and modelled (dashed line) time series.

The results of the Bayesian modelling for each outcome are shown in Table 1 and graphically in Fig. 3 and supplementary Fig. 1. Comparing the counterfactual trends to the actual trends observed in the post-investment period, indicated that the median waiting time from arriving in the emergency department to receiving a psychosocial assessment was approximately 2 h shorter per month (18.6% decrease, 95% BCI −30.2% to −2.8%), although this effect appears to be diminishing with time (Fig. 3(e)). The mean cost of a psychosocial assessment increased by about £18 (10.3% increase, 95% BCI 5.3% to 15.7%) and mean emergency department costs reduced by £1.50 per episode (0.8% decrease, 95% BCI −2.4% to 0.4%). There was little evidence of an effect on mean observation ward costs (8.7% increase, 95% BCI −12.0% to 35.3%), mean ITU costs (4.6% decrease, 95% BCI −120.5% to 129.2%) and mean net hospital costs (5.3% increase, 95% BCI −11.6% to 25.5%).

Time series for (a) the number of episodes admitted to a hospital ward; (b) the number of episodes with a psychosocial assessment; (c) the number of episodes self-discharging from the emergency department without an assessment; (d) the number of patients with repeat emergency department attendances within 6 months from index attendance; (e) the median waiting time from emergency department arrival to assessment (hours); (f) mean net hospital costs. Shaded areas correspond to 95% credible intervals.

**Table 1:** Inferred effect of the September 2014 investment on 3-year average outcomes

Outcome	Relative average effect, % (95% BCI)	Absolute average monthly effect of the investment	Posterior predictive *P*
Episode outcomes			
Number of episodes admitted to intensive therapy unit	−28.3 (−49.5 to −6.8)	−0.5 episodes	0.008
Number of episodes admitted to a hospital ward	0.1 (−4.3 to 3.6)	+0.1 episodes	0.449
Number of referrals made to other agencies	86.1 (60.6 to 110.9)	+45.2 referrals	<0.001
Number of episodes self-discharging from the emergency department without an assessment	−7.7 (−21.6 to 5.5)	−0.9 episodes	0.102
Psychosocial assessments			
Number of episodes with a psychosocial assessment	11.7 (−3.4 to 28.5)	+6.9 episodes	0.043
Median waiting time from emergency department arrival to assessment, h	−18.6 (−30.2 to −2.8)	−2.1 h	0.014
Repeat attendances			
Number of patients with repeat emergency department attendances within 6 months from index date	−11.2 (−39.6 to 27.3)	−0.8 patients	0.198
Median time to first repeat attendance, days	28.6 (−32.6 to 104.9)	+12.2 days	0.186
Cost outcomes			
Mean psychosocial assessment costs	10.3 (5.3 to 15.7)	+£18.3 per assessment	<0.001
Mean emergency department costs	−0.8 (−2.4 to 0.4)	−£1.5 per episode	0.092
Mean observation ward costs	8.7 (−12.0 to 35.3)	+£17.3 per episode	0.183
Mean intensive therapy unit costs	−4.6 (−120.5 to 129.2)	−£3.4 per episode	0.439
Mean net hospital costs	5.3 (−11.6 to 25.5)	+£33.6 per episode	0.261

Following the 2014 investment there were indications of an increase in the number of episodes that received a psychosocial assessment (11.7% increase, 95% BCI −3.4% to 28.5%) and a decrease in the number of episodes self-discharging from the emergency department without a psychosocial assessment (7.7% decrease, 95% BCI −21.6% to 5.5%), however, the evidence is inconclusive.

There were approximately 45 referrals more per month to other agencies (86.1% increase, 95% BCI 60.6% to 110.9%) and there was little evidence that median time to first repeat attendance had changed (12 days longer per month, 28.6% increase, 95% BCI −32.6% to 104.9%).

All models produced stable estimates and demonstrated acceptable convergence for the majority of variables after 100 000 Markov chain Monte Carlo samples. Comparison of the observed and modelled data during the pre-investment period indicated that the observed time series were accurately modelled, with a mean absolute 1-step prediction error less than 10% of the pre-investment outcome mean for the majority of outcomes (64%) and between 10% and 30% for the remainder. As the assumption that there is no change in correlations between the outcomes and covariates post-investment is correct then the counterfactual time series is expected to be of similar accuracy.

## Discussion

### Main findings

Following the 2014 investment, there was a decrease in the waiting time from emergency department arrival to receiving a psychosocial assessment, a considerable increase in the number of referrals to other agencies and an increase in the absolute number of psychosocial assessments. Evaluation of costs showed an increase in average cost of a psychosocial assessment, however, there was little evidence of an overall increase in mean net hospital costs.

### Comparison with other studies

UK clinical guidelines recommend psychosocial assessment for all patients presenting with self-harm.^13^ The findings of this evaluation suggest that the short-term decrease in median waiting time from arrival in emergency department to psychosocial assessment identified by Opmeer *et al*^18^ has persisted longer term, with a median 2 h reduction in waiting times. Short-term increases in referrals to the crisis or other community teams also appear to have continued, with an overall increase in referrals made to other agencies identified long term. In contrast, evidence for a reduction in the number of episodes self-discharging without an assessment was less clear than in the initial evaluation.^18^ There was an indication of an increase in the number of episodes that received a psychosocial assessment, however, this finding was also less clear than in the initial evaluation. Neither evaluation identified an effect on the number of patients with repeat self-harm attendances or median time to first repeat following the investment.

Economic evaluation of the RAID (rapid assessment interface and discharge) LPS at a large acute hospital in Birmingham reported cost savings based on reduced length of stay, avoided admissions to general wards and reduced rates of readmission, with the majority of savings as a result of shorter lengths of stay among older patients (>65 years).^17^ Less than 28% of referrals to RAID were for self-harm, however, whereas the current evaluation focused solely on LPS activities for patients who have self-harmed who comprise only 40% of the liaison team workload.

### Interpretation of results

Despite the annual increase in the number of attendances for self-harm the number (and proportion) of episodes receiving a psychosocial assessment increased and the number of episodes self-discharging without an assessment fell, indicating the resilience of the LPS in face of mounting pressures. Patient's risk is assessed at triage using a risk matrix, with higher-risk patients prioritised for psychosocial assessment. Further research is required to understand the reasons underlying instances of non-assessment of patients referred for psychosocial assessment, but may include self-discharge, patient's refusal, low-risk score, under existing psychiatric care.^31^ The diminishing effect of the investment on waiting time from emergency department arrival to receiving psychosocial assessment demonstrates a continuing need for investment in order to meet service demands in light of the annual increases in attendances. The small (5%) increase in net costs identified in this evaluation is most likely a reflection of the increase in the proportion of psychosocial assessments carried out by liaison nurses that are associated with a higher cost than assessments carried out by a doctor (often trainee doctors). The inferred increase in referrals to other agencies is possibly a reflection of improved risk assessment and patient management following the investment, potentially as a result of the increased capacity of liaison nurses, who are generally more experienced than trainee doctors in the emergency department and likely to have a more detailed knowledge of potential local referral agencies. The increase in psychosocial assessment costs may be offset by such improvements in the management of patients who self-harm and referral to appropriate services if these result in improved patient outcomes.

### Strengths

The methods used in the current study not only enabled the evaluation of the effects of the intervention over a longer, 3-year period of time than the previous evaluation by Opmeer et al,^18^ but was also able to account for secular and seasonal trends. In addition, in the absence of randomised controls, the Bayesian structural time series method enabled the construction of a synthetic control to act as a comparison with the observed data. Finally, Bayesian model averaging minimises issues of arbitrary and incorrect covariate selection and of overfitting.^20^

Compared with previous studies based on select groups of patients (such as those who had taken an overdose)^32^ or on small numbers of patients,^11^ the data used in this analysis was taken from an unselected series of consecutive presentations of self-harm, including those that were admitted to hospital as well as those with emergency department attendance that did not result in admission.

### Limitations

An important limitation is that unobserved variables might have improved the counterfactual estimation, thereby reducing the potential of bias. Through the use of Bayesian model averaging in combination with spike and slab priors inclusions of the covariates were weighted to avoid overfitting, but we cannot exclude that other variables, not *a priori* selected, could have improved model fit and may have further improved forecasting accuracy.

Despite several methodological advantages of the use of this Bayesian framework, a limitation is that it requires more data than non-Bayesian methods to obtain comparable forecasting precision and to adequately model the counterfactual. The relatively wide credible intervals around important outcomes, such as re-attendance for self-harm, indicate that a larger study would be required to provide stronger evidence.

As noted by Opmeer et al,^18^ of all LPS referrals, psychosocial assessment of people attending emergency department for self-harm comprises only 40%. Although the expansion of the LPS operating hours is likely to result in a better service for all patients with psychiatric morbidity, neither the short-term nor long-term evaluations measured the impact of other (non-self-harm related) activities of the liaison psychiatry team at BRI. Furthermore, we were unable to assess the full economic impact of the LPS on NHS costs as the BSHSR does not record details of subsequent primary and community care services. The increase in referrals to other agencies could plausibly either increase or decrease the long-term costs of care for patients who have self-harmed.

## Data Availability

Data associated with this manuscript is accessible only to the research team and is not publicly available

## Supplementary material

For supplementary material accompanying this paper visit http://dx.doi.org/10.1192/bjo.2020.18. click here to view supplementary material
