# Supplementary material for: Long-term impact of the expansion of a hospital liaison psychiatry service on patient care and costs following emergency department attendances for self-harm
Source: BJPsych Open. 2020 Apr 2;6(3):e34. doi: 10.1192/bjo.2020.18 (PMC7176831; doi:10.1192/bjo.2020.18)
Supplement: Supplementary file 1 [file S2056472420000186sup001.docx]

**Supplementary materials**

**Table S1. Cost breakdown of psychosocial assessments carried out by a Liaison Nurse, Doctor, Joint or other/unknown professional**

|  |  | **Liaison nurse (band 7)** | FY1 | FY2 | Junior (average FY1 & FY2 costs) | Registrar | Psychiatric consultant | Senior (average registrar and consultant costs) | **Doctor (weighted 80:20 average of junior and senior doctor costs)** | **Joint costs** | **Other/unknown professional** |
| --- | --- | --- | --- | --- | --- | --- | --- | --- | --- | --- | --- |
| Working hours per year | | **1573** | 2,138 | 2,138 | 2,138 | 2138 | 1842 | 1,990 | **2,108** | **3,681** | **1,841** |
| Salary | | **£39,039** | £26,635 | £30,354 | £28,495 | £41,583 | £91,926 | £66,755 | **£36,147** | **£75,186** | **£37,593** |
| Salary oncosts | | **£9,875** | £6,379 | £7,427 | £6,903 | £10,591 | £24,778 | £17,685 | **£9,059** | **£18,934** | **£9,467** |
| Overheads | |  |  |  |  |  |  |  |  |  |  |
|  | Management, admin and estates staff | **£11,837** | £7,989 | £9,143 | £8,566 | £12,626 | £28,242 | £20,434 | **£10,940** | **£22,777** | **£11,388** |
|  | Non-staff | **£21,082** | £14,229 | £16,284 | £15,257 | £22,487 | £50,299 | £36,393 | **£19,484** | **£40,566** | **£20,283** |
| Capital overheads | | **£3,462** | £4,710 | £4,710 | £4,710 | £4,710 | £6,115 | £5,413 | **£4,851** | **£8,313** | **£4,156** |
| Total cost | | **£85,295** | £59,942 | £67,918 | £63,930 | £91,997 | £201,360 | £146,679 | **£80,480** | **£165,775** | **£82,887** |
| Indirect time (1:1.44) | | **£122,825** | £86,316 | £97,802 | £92,059 | £132,476 | £289,958 | £211,217 | **£115,891** | **£238,716** | **£119,358** |
| Total cost including indirect time | | **£208,120** | £146,258 | £165,720 | £155,989 | £224,473 | £491,318 | £357,896 | **£196,370** | **£404,490** | **£202,245** |
| **Psychosocial assessment cost (90 minutes)** | | **£198** | £103 | £116 | £109 | £157 | £400 | £279 | **£143** | **£341** | **£170.5** |

**Model specifications**

Bayesian priors were specified as: prior standard deviation and initial values: 20% of the observed pre-investment period standard deviation (allowing the modelled time series freedom, whilst retaining predictive power); upper limit for the Inverse Gamma standard deviation: 150% of the observed pre-investment period standard deviation. The stationary distribution of the AR(1) process was used as the initial state distribution, describing values at time 0. A spike-and-slab prior was placed on the regression coefficients of the covariates, set to expect that the model would be informed by the 3 covariates described above, with priors for the effect sizes set to a mean of ‘0’ (no effect) with the exception of the intercept which was set to the outcome mean. The standard deviation of the regression coefficients was also set to 20% of the observed pre-investment period standard deviation and the prior expected explained variance set to 70% with 35 degrees of freedom. Prior inclusion probabilities were not provided for any of the covariates, making their inclusion data driven. Counterfactual timeseries were based on marginal inclusion probabilities of each regression coefficient through Bayesian model averaging. Model fit was assessed using the following post-hoc tests: Geweke diagnostics (convergence of MCMC chains), Raftery-Lewis diagnostic tests (mixing, correlation and inappropriate starting values), precision (using mean absolute 1-step prediction errors) and Durbin-Watson, Ljung-Box tests and autocorrelation plots (residual correlation). Sensitivity analyses with different priors did not materially change the results. Additional sensitivity analysis was conducted to check whether inclusion of a local linear trend that would indicate a continuing in/decrease in the outcome over the complete time-period could improve the models. There was no evidence that inclusion changed any of the conclusions, however there was insufficient data to include this parameter.

**Table S2. Covariates and outcomes before and after the investment in September 2014**

| **Covariates** | **Pre investment period, mean (range)*** | **Post investment period, mean (range)*** | **Total over entire study period, mean (range)*** |
| --- | --- | --- | --- |
| **Number of attendances for self-harm** | 91.91 (66.00 - 130.00) | 103.79 (72.00 - 139.00) | 98.10 (66.00 - 139.00) |
| **Mean age of patients attending for self-harm** | 33.38 (31.36 - 36.11) | 33.38 (29.33 - 36.83) | 33.38 (29.33 - 36.83) |
| **Proportion of females attending for self-harm** | 57.60 (43.66 - 67.50) | 63.28 (42.55 - 78.00) | 60.56 (42.55 - 78.00) |
| **Episode outcomes** | **Pre investment period** | **Post investment period** | **Total** |
| **Number of episodes admitted to Intensive Therapy Unit (ITU)** | 1.71 (0.00 - 5.00) | 1.24 (0.00 - 5.00) | 1.47 (0.00 - 5.00) |
| **Number of episodes admitted to a hospital ward** | 60.69 (47.00 - 85.00) | 66.11 (48.00 - 92.00) | 63.51 (47.00 - 92.00) |
| **Number of referrals made to other agencies** | 47.11 (28.00 - 88.00) | 97.63 (46.00 - 191.00) | 73.41 (28.00 - 191.00) |
| **Number of episodes self-discharging from the ED without an assessment** | 11.20 (4.00 - 20.00) | 10.58 (3.00 - 22.00) | 10.88 (3.00 - 22.00) |
| **Psychosocial assessments** |  |  |  |
| **Number of episodes with a psychosocial assessment** | 52.97 (37.00 - 85.00) | 65.84 (45.00 - 90.00) | 59.67 (37.00 - 90.00) |
| **Median waiting time from ED arrival to assessment (hours)** | 11.57 (6.92 - 14.27) | 9.02 (6.34 - 11.98) | 10.25 (6.34 - 14.27) |
| **Repeat attendances** |  |  |  |
| **Number of patients with repeat ED attendances within 6 months from index date** | 7.20 (2.00 - 15.00) | 6.00 (1.00 - 12.00) | 6.63 (1.00 - 15.00) |
| **Median time to first repeat attendance (days)** | 51.09 (2.00 - 110.00) | 54.89 (7.00 - 162.00) | 53.07 (2.00 - 162.00) |
| **Cost outcomes** |  |  |  |
| **Mean psychosocial assessment costs** | 181.30 (169.60 - 199.70) | 195.70 (178.70 - 227.30) | 188.80 (169.60 - 227.30) |
| **Mean ED costs** | 189.40 (176.90 - 195.70) | 187.50 (178.70 - 198.80) | 188.40 (176.90 - 198.80) |
| **Mean observation ward costs** | 213.40 (135.70 - 452.10) | 215.80 (125.00 - 412.40) | 214.60 (125.00 - 452.10) |
| **Mean ITU costs** | 112.19 (0.00 - 357.34) | 70.36 (0.00 - 371.49) | 90.41 (0.00 - 371.49) |
| **Mean net hospital costs** | 696.30 (531.40 - 1,058.20) | 669.30 (497.90 - 1,082.70) | 682.30 (497.90 - 1,082.70) |

* Summarised from monthly frequency / average / proportion


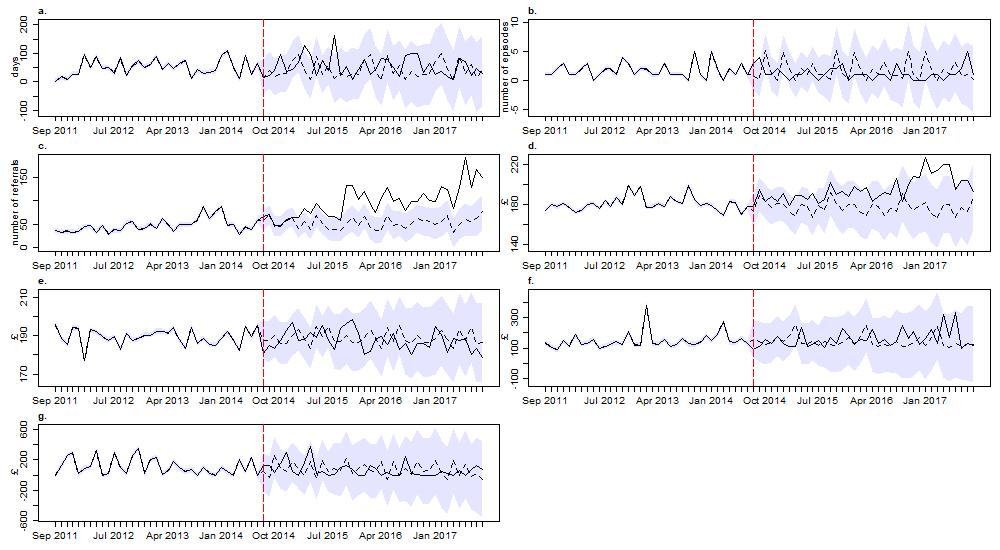


**Figure S1**. Observed (solid line) and modelled (dashed line) time series for a. median time to first repeat attendance (days), b. number of episodes admitted to ITU, c. number of referrals to other agencies, d. mean psychosocial assessment costs, e. mean ED costs, f. mean observational ward costs, g. mean ITU costs. Shaded areas correspond to 95% Credible Intervals
